# Detection of exercise intensity thresholds in patients with chronic heart failure based on correlation properties of heart rate variability

**DOI:** 10.1007/s00421-025-05860-9

**Published:** 2025-06-25

**Authors:** Noemí Sempere-Ruiz, Agustín Manresa-Rocamora, Laura Fuertes-Kenneally, Ana Sanz-Rocher, Sabina Baladzhaeva, Vicente Climent-Payá, Manuel Moya-Ramón, José M. Sarabia

**Affiliations:** 1 Department of Sport Sciences, Sports Research Centre, Miguel Hernández University of Elche, 03202 Elche, Spain; 2 Institute for Health and Biomedical Research of Alicante (ISABIAL), 03010 Alicante, Spain; 3 Cardiology Department, Dr. Balmis General University Hospital, 03010 Alicante, Spain

**Keywords:** Aerobic exercise, Cardiac rehabilitation, Autonomic nervous system, DFA a1, Ventilatory thresholds, Cardiopulmonary exercise test

## Abstract

**Purpose:**

This study aimed to assess the agreement between ventilatory thresholds (VT1 and VT2), and heart rate variability (HRV) thresholds (HRVT1 and HRVT2) based on the alpha 1 index of detrended fluctuation analysis (DFA a1) in patients with chronic heart failure (CHF). Validating HRV-based thresholds could provide a cost-effective alternative for individualised exercise intensity prescription, improving safety and efficacy in exercise-based cardiac rehabilitation (CR) programmes.

**Methods:**

Twenty CHF patients (13 males, 7 females) performed a cardiopulmonary exercise test (CPET) on a cycle ergometer. Ventilatory thresholds were identified using a mixed method, while HRV thresholds were determined at DFA a1 values of 0.75 (HRVT1) and 0.5 (HRVT2). Threshold values for oxygen consumption (VO_2_), heart rate (HR), and power output (PO) were compared with paired *t* test or Wilcoxon test. Agreement was assessed using correlation coefficients (Pearson’s *r* and Spearman’s *rho*), intraclass correlation coefficient (ICC), and Bland–Altman analysis.

**Results:**

HRVT2 showed moderate-to-strong associations with VT2 for VO_2_ (*rho* = 0.88, ICC = 0.86), for HR (*r* = 0.88, ICC = 0.81) and for PO (*r* = 0.82, ICC = 0.85). Mean biases were small and limits of agreement (LoA) narrow. HRVT1 correlated only modestly with VT1 for VO_2_ (*rho* = 0.67, ICC = 0.43) and weakly for HR (*r* = 0.43, ICC = 0.37) and PO (*r* = 0.49, ICC = 0.35), with wide LoA.

**Conclusion:**

In CHF patients, HRVT2 appears to be a valid, practical surrogate for VT2 and may facilitate personalised intensity prescription where full CPET is unavailable. HRVT1 showed insufficient agreement with VT1 and should be used with caution. Larger cohorts and protocol refinements are warranted to confirm these observations and to explore strategies for improving HRVT1 accuracy.

**Graphical Abstract:**

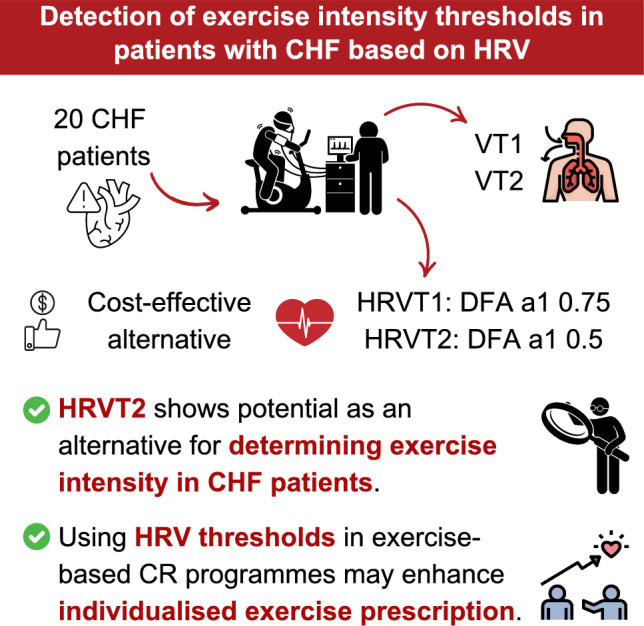

## Introduction

Chronic heart failure (CHF) is one of the most prevalent life-threatening conditions worldwide, with a prevalence estimated at approximately 2% among adults in developed countries, rising to over 10% in individuals aged 70 years or older (Van Riet et al. 2016). Moreover, in contrast to other cardiovascular diseases such as coronary artery disease or stroke, its incidence and prevalence have increased in recent years (Zannad 2018), underscoring the urgent need to improve prevention and treatment strategies to enhance diagnosis, quality of life, and clinical outcomes. Among these strategies, exercise-based cardiac rehabilitation (CR) programmes have demonstrated robust efficacy in improving functional capacity, reducing symptoms, and lowering mortality rates in patients with CHF (Tegegne et al. 2022). Thus, exercise-based CR is considered a cornerstone of comprehensive CHF management.

Aerobic exercise, particularly performed at moderate-intensity, has traditionally been the main modality employed in exercise-based CR programmes due to its well-documented benefits, including improved cardiorespiratory fitness, an independent mortality predictor (Sarabia et al. 2018; Hansen et al. 2022). However, despite their effectiveness, high heterogeneity exists in individual patient responses (Iannetta et al. 2023). These findings highlight the need for more precise aerobic exercise prescription methods to reduce the prevalence of non-responders and optimise outcomes (Inglis et al. 2024). In this context, accurate determination of aerobic exercise intensity thresholds is critical to achieve the desired metabolic and physiological adaptations in patients with CHF.

Exercise thresholds represent key physiological transition points, reflecting shifts in energy metabolism and cardiovascular response. Their accurate identification is particularly important for optimising training programmes for individuals with CHF (Keir 2024). Gas exchange analysis is commonly used to determine the first and second ventilatory thresholds (VT1 and VT2, respectively). Ventilatory thresholds serve as non-invasive proxies for the underlying metabolic landmarks—VT1 closely approximates the lactate threshold, whereas VT2 corresponds to the upper boundary of the heavy-intensity domain (maximal metabolic steady state), marking critical metabolic and ventilatory transitions during exercise (Keir et al. 2022). Specifically, the cardiopulmonary exercise test (CPET) with gas exchange analysis is considered the gold standard for determining these thresholds; however, its reliance on costly equipment and specialised personnel limits its widespread implementation. This underscores the need for alternative, cost-effective methods to determine exercise intensity thresholds and improve aerobic exercise prescription in exercise-based CR settings.

Heart rate (HR) variability (HRV) has emerged as a promising, non-invasive tool for evaluating exercise capacity and determining exercise intensity thresholds (Kaufmann et al. 2023). HRV quantifies the variation in time intervals between heartbeats (RR intervals) and reflects autonomic nervous system (ANS) activity (Gronwald and Hoos 2020). Notably, HRV can be measured using any device that records RR intervals, such as HR monitors or electrocardiography (ECG) systems, making it an accessible tool for clinical and field-based assessments (Rogers et al. 2021a). Among the methods used to determine HRV-based thresholds (HRVT), the short-term scaling exponent alpha 1 from detrended fluctuation analysis (DFA a1) has gained particular attention for its potential to non-invasively assess exercise intensity distribution (Gronwald et al. 2020).

The DFA a1 index offers a cost-effective measure of ANS response to exercise, reflecting systemic stress and homeostatic balance (Rogers and Gronwald 2022). DFA a1 values typically range from values of 1.5 at rest or during low-intensity exercise, down to 0.5 or even lower as exercise intensity increases. A DFA a1 value of 1.5 indicates well-regulated physiological control with correlated behaviour, while a value of 0.5 or below represents a chaotic state of physiological regulation, indicating that the body is struggling to maintain balance under increased stress and showing more random or anticorrelated patterns (Gronwald and Hoos 2020). This behaviour has led to the establishment of absolute thresholds of 0.75 (referred to as HRVT1) and 0.5 (referred to as HRVT2), which have been associated with VT1 and VT2, respectively, in different populations (Rogers et al. 2021b, c, 2022; Schaffarczyk et al. 2022; Sempere-Ruiz et al. 2024). In this regard, a recent study demonstrated a strong correlation between HRVT1 and VT1 in males with cardiac disease; however, it did not differentiate between coronary artery disease and congestive heart failure (Rogers et al. 2021d). On the contrary, no studies to date have explored the agreement between HRVT2 and VT2 in patients with CHF. Given the altered autonomic regulation in CHF patients (Dagres and Hindricks 2013), HRV responses to exercise may differ significantly from those observed in other groups. This underscores the need to validate HRVT specifically in CHF patients to ensure their reliability and clinical utility.

Thus, this study aims to determine the agreement between ventilatory (i.e., VT1, VT2) and HRV (i.e., HRVT1, HRVT2) thresholds using the DFA a1 method in patients with CHF. Based on prior work in cardiac populations, we hypothesised that (i) the first HRV threshold (HRVT1, DFA a1 = 0.75) would exhibit a strong agreement with the first ventilatory threshold (VT1), and (ii) the second HRV threshold (HRVT2, DFA a1 = 0.5) would demonstrate at least a moderate agreement with the second ventilatory threshold (VT2) in patients with CHF. The findings are expected to contribute to more precise and individualised aerobic exercise prescription strategies, ultimately enhancing the safety and efficacy of exercise-based CR programmes. The integration of DFA a1 into CR may enable better monitoring of patient progress, paving the way for safer and more effective exercise-based CR interventions.

## Method

### Patients

Participants were recruited from the General University Hospital Dr. Balmis on a voluntary basis through a telephone call. Inclusion criteria were: (a) male and female patients diagnosed with CHF (Bozkurt et al. 2021) who had stable symptoms, therapy, and medical treatment for at least the last 3 months, regardless of the left ventricular ejection fraction (LVEF); (b) aged ≥ 18 years; (c) ischaemic and non-ischaemic aetiology; (d) New York Heart Association functional class II–III; and (e) no physical limitations to exercise. Exclusion criteria were: (a) unstable angina pectoris; (b) myocardial infarction over the past 6 months; (c) chronic obstructive pulmonary disease; and (d) complex ventricular arrhythmias. Inclusion and exclusion criteria were carefully checked by experimented cardiologists.

### Study design

The study protocol was approved by the Ethics Committee of the hospital (Ref: 2022-140; date of approval: 13/01/2023). Patients who met the inclusion criteria were asked to attend the hospital in the afternoon (between 16:00 p.m. and 19:00 p.m.). No strenuous exercise was allowed the day before (i.e., 24 h) the visit to the hospital. Before starting, patients were informed about the study and those who agreed to participate in the investigation provided a written informed consent. Anthropometrical variables were measured (i.e., weight, height, and body mass index [BMI]), and a CPET was conducted as explained below.

### Cardiopulmonary exercise test

All patients carried out a symptom-limited CPET on a cycle ergometer (SanaBike 500 easy, Truchtelfinger, Germany) under medical supervision. Patients who were unable to complete the CPET (i.e., symptom-limited CPET) were excluded from the analysis. The CPET consisted of the following phases: (a) a 5-min resting period in the seated position on the cycle ergometer; (b) a 3-min warm-up at 10 W; (c) an individualised ramp protocol was employed, with slope increments adjusted between 4 and 15 W·min^−1^ according to each patient’s predicted VO_2_ peak, aiming for volitional exhaustion within 8–12 min (Manresa-Rocamora et al. 2023), and a pedalling frequency set between 60 and 70 rpm; and (d) a 5-min cool-down at 10 W.

The respiratory gas exchange and HR were monitored continuously during the full exercise test by Metalyzer 3B (CORTEX Biophysik GmbH, Leipzig, Germany) and a 12-lead ECG (Norav PC-ECG 1200, Mainz-Kastel, Germany), respectively. The ECG electrodes were placed in standard position and filters for 50 Hz and electromiography (i.e., 35 Hz) were activated. Ventilatory data were averaged every 10 s.

### Calculation of ventilatory thresholds

Ventilatory thresholds were identified using a combined method to enhance the reliability of the determinations, as recommended by Keir et al. (2022). Ventilatory data were plotted and reviewed independently by two experienced researchers (AM and LF) to identify VT1 and VT2 values. In case of discrepancies, a third experienced researcher (JMS) conducted a blind analysis to establish a consensus. For each threshold, values of oxygen uptake (VO_2_), HR, and power output (PO) were determined. HR and PO at VT1 and VT2 were derived using temporal interpolation.

### Calculation of heart rate variability thresholds

Sample data from the ECG were imported to Kubios HRV Scientific 4.0.1 (Biosignal Analysis and Medical Imaging Group, Department of Physics, University of Kuopio, Kuopio, Finland). Kubios HRV preprocessing settings adhered to default values, which included utilising the RR detrending method “Smoothn priors” with a lambda value of 500 (Tarvainen et al. 2014). The automatic beat correction filter was applied, and participants who exhibited more than 5% of artifacts in their data were excluded from the subsequent analysis. For the estimation of DFA a1, it was measured the root mean square fluctuation of the integrated and detrended data within observation windows of varying sizes. The data were then plotted on a log–log scale, with the scaling exponent representing the slope of the line that relates the (log) fluctuation to the (log) window size (Mendonca et al. 2010). The window width for DFA a1 analysis was set to a range of 4 to 16 beats.

DFA a1 values of 0.75 and 0.5 were selected for HRVT1 and HRVT2, respectively (Rogers et al. 2021d, c). DFA a1 was computed from the RR series collected during the incremental exercise test using 2-min windows, with recalculations performed every 5 s throughout the test. To determine HR, VO_2_, and PO at HRV thresholds, the method outlined in previous studies was applied (Rogers et al. 2021b). Specifically, for HR data, DFA a1 was plotted against HR, and a linear regression was performed for values ranging from about 1.0 to 0.5. For VO_2_ and PO, DFA a1 was plotted against time, using the same approach. The HR or time points where DFA a1 reached 0.75 and 0.5 were identified through the linear regression equation. The corresponding time points were converted into VO_2_ and PO values using their respective linear regression models for VO_2_ vs time and PO vs time.

### Statistical analyses

The Shapiro–Wilk test and box plots were used to verify the normal distribution of the continuous variables. Normal and non-normal distributed data were presented as mean ± standard deviation (*SD*) or median (25th and 75th percentiles), respectively. Moreover, categorical data were reported as frequency (percentage). The values obtained at the first and second thresholds using the two methods were compared with the paired *t* test and Wilcoxon signed-rank test for parametric and non-parametric data, respectively. In addition, mean or median difference with its 95% confidence interval (95%CI) were estimated. Agreement between the two methods (i.e., VT and HRVT) in determining HR, VO_2_, and PO at first and second thresholds was assessed using Pearson’s *r* correlation and Spearman’s *rho* correlation coefficient for parametric and non-parametric data, respectively. The strength of the correlation was interpreted as weak (< 0.30), low (0.30–0.49), moderate (0.50–0.69), strong (0.70–0.89), and very strong (> 0.90) (Pett 2016). Additionally, the intraclass correlation coefficient (ICC) and Bland–Altman analysis with limits of agreement were obtained. ICC values were interpreting as poor (< 0.5), moderate (0.5–0.75), good (0.75–0.9), and excellent reliability (> 0.9). Statistical significance was set at *p* ≤ 0.05. JASP v0.18.1.0 (JASP Team 2019; jasp-stats.org) and Microsoft Excel 365 for Windows were used for conducting the analyses.

## Results

Forty-two patients with CHF met the inclusion criteria and were invited to participate in the current study. Nonetheless, 22 (52.4%) patients were excluded due to the following reasons: (a) symptom-limited CPET (*n* = 3); (b) failure to identify first and second thresholds using the one or both methods (i.e., VT or/and HRVT) (*n* = 12), and (c) HR data artifacts exceeding 5% (*n* = 7). Consequently, 20 (47.6%) patients were included in the final analysis, whose demographic and clinical characteristics are shown in Table 1.

**Table 1:** Demographic and clinical characteristics

Characteristic	All patients (*n* = 20)
Sex (male/female)	13 (65)/7 (35)
Age, years	59 ± 7 (41–73)
Weight, kg	80.6 ± 19.5 (42.5–137.0)
Height, cm	167.0 ± 9.7 (151.0–185.0)
BMI, kg/m^2^	28.6 ± 5.0 (17.2–40.9)
LVEF, %	40.2 ± 8.5 (26.4–59.7)
VO_2_ peak	18.4 (16.4–20.1) (12.9–32.8)
HR peak	129 ± 20 (81–158)
PO peak	91 ± 37 (36–161)
Smoker (yes/no)	13 (65)/7 (35)
Hypertension (yes/no)	8 (40)/12 (60)
Diabetes (yes/no)	7 (35)/13 (65)
Dyslipidaemia (yes/no)	9 (45)/11 (55)
ICD (yes/no)	8 (40)/12 (60)
b-Blockers (yes/no)	13 (65)/7 (35)
ACE inhibitors (yes/no)	7 (35)/13 (65)
MRA (yes/no)	11 (55)/9 (45)
Antiplatelets (yes/no)	2 (10)/18 (90)
Diuretics (yes/no)	6 (30)/14 (70)

*ACE* angiotensin-converting enzyme, *BMI* body mass index, *HR peak* heart rate peak, *ICD* implantable cardioverter defibrillator, *LVEF* left ventricular ejection fraction, *MRA* mineralocorticoid receptor antagonists, *PO peak* power output peak, *VO*_*2*_* peak* peak oxygen uptake

Values are reported as mean ± standard deviation or median (25th–75th percentiles) (minimum and maximum values), and frequency (percentage)

### Comparison between methods

The HR, VO_2_, and PO at VT1, VT2, HRVT1, and HRVT2 are shown in Table 2. Comparisons, correlations, ICC, and Bland–Altman values between values obtained using the ventilatory and HRV threshold methods are presented in Table 3.

**Table 2:** Descriptive data of ventilatory and HRV thresholds

	VT1	VT2	HRVT1	HRVT2
HR (bpm)	95 ± 10	113 ± 16	102 ± 18	112 ± 20
HR (% HR peak)	74.5 ± 9.9	88.0 ± 5.2	79.0 ± 9.8	86.4 ± 9.2
PO (W)	40 ± 15	73 ± 30	51 ± 36	67 ± 34
PO (% PO peak)	45 ± 9	81 ± 8	55 ± 25	74 ± 22
VO_2_ abs* (l·min^−1^)	0.9 (0.8–1.0)	1.3 ± 0.4	1.1 ± 0.4	1.2 ± 0.4
VO_2_ rel* (ml·kg^−1^·min^−1^)	11.0 (10.1–12.3)	14.8 (13.5–16.3)	11.9 (10.3–13.8)	14.0 (12.4–17.3)
VO_2_* (% VO_2_ peak)	60.8 ± 9.1	82.7 ± 5.7	70.3 (63.9–75.7)	79.5 ± 11.7

*HR* heart rate, *HRVT1* first heart rate variability threshold, *HRVT2* second heart rate variability threshold, *PO* power output, *VO*_*2*_ oxygen uptake, *VT1* first ventilatory threshold, *VT2* second ventilatory threshold, *Normal distributed data are reported as mean ± standard deviation, and non-normal distributed data are reported as median (25th–75th percentiles)

**Table 3:** Comparison of ventilatory and HRV thresholds in HR, VO_2_, and PO values

	Comparison	Mean/median difference			Correlation coefficient		ICC_3,1_		Bland–Altman	
		Difference	95% CI	*p* value	*r/rho* value	*p* value	Value	95% CI	Bias	SD
HR (bpm)	HRVT1–VT1	7	− 1–14	0.08	0.43	0.06	0.37	− 0.08–0.70	7	16
	HRVT2–VT2	− 2	− 6–3	0.47	0.88	< 0.01	0.85	0.66–0.94	− 2	10
PO (W)	HRVT1–VT1	11	− 3–25	0.12	0.49	0.03	0.35	− 0.10–0.68	11	31
	HRVT2–VT2	− 6	− 15–3	0.20	0.82	< 0.01	0.81	0.59–0.92	− 6	20
VO_2_* (ml·kg^−1^·min^−1^)	HRVT1–VT1	1.3	− 0.2–3.5	0.11	0.67	< 0.01	0.43	− 0.00–0.73	1.8	4.1
	HRVT2–VT2	− 0.7	− 1.6–0.7	0.37	0.88	< 0.01	0.86	0.69–0.94	− 0.5	2.4

*95% CI* ICC confidence interval, *HR* heart rate, *HRVT1* first heart rate variability threshold, *HRVT2* second heart rate variability threshold, *ICC* intraclass correlation coefficient, *SD* standard deviation, *PO* power output, *VO*_*2*_ oxygen uptake, *VT1* first ventilatory threshold, *VT2* second ventilatory threshold. *Non-normal distributed data

**Fig. 1:**
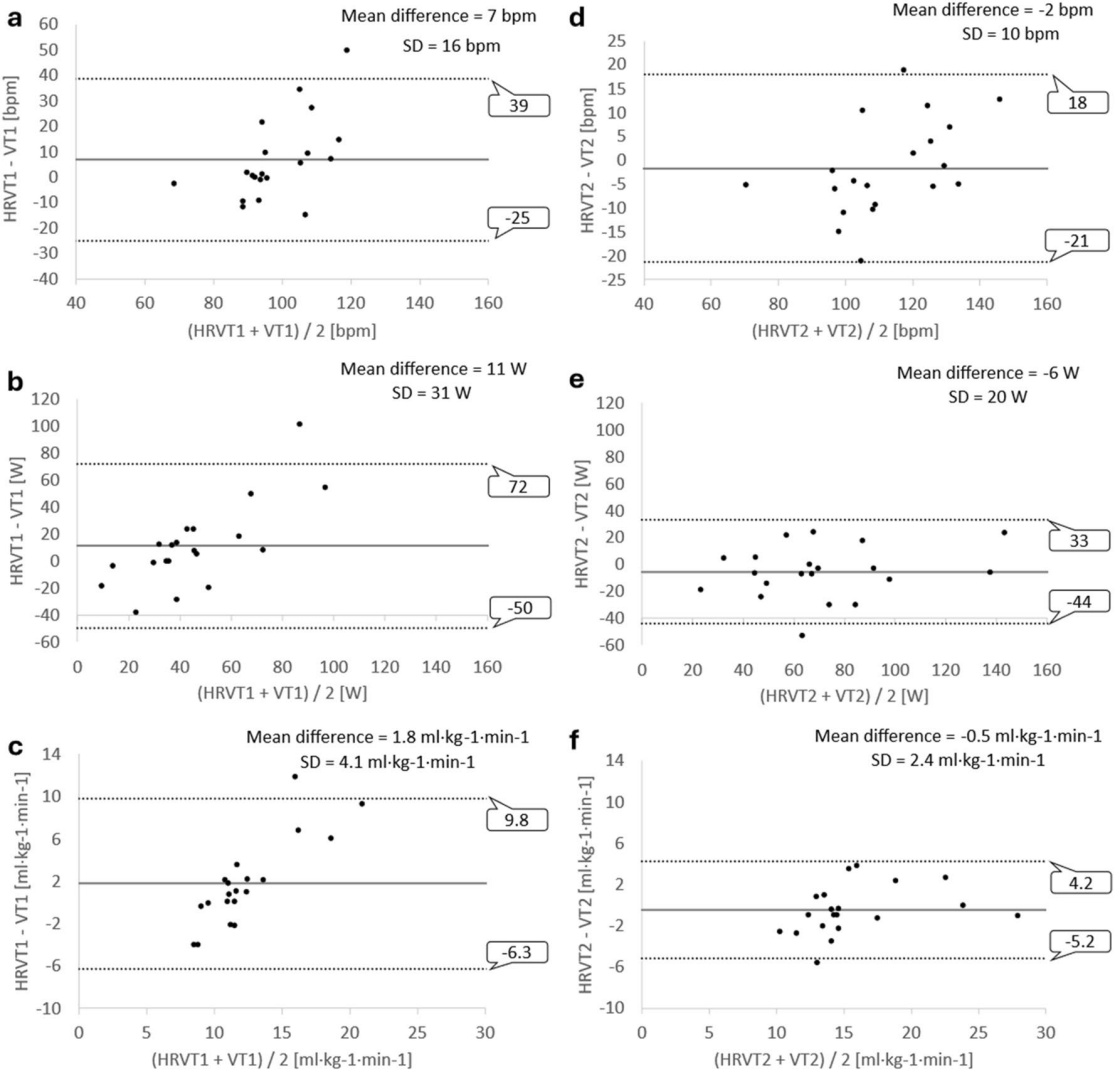
Bland–Altman plots for the comparison between HRVT and VT measured in (**a**, **d**) HR (bpm) values; (**b**, **e**) PO (W) values, and (**c**, **f**) VO_2_ (ml·kg^−1^·min^−1^) values

Regarding the first threshold, VO_2_ values showed a moderate correlation (*rho* = 0.67, *p* < 0.01), whereas HR (*r* = 0.43, *p* = 0.06) and PO (*r* = 0.49, *p* = 0.03) exhibited low correlations between HRVT1 and VT1. No statistically significant mean differences were found for HR (7 bpm, 95% CI: − 1–14), PO (11 W, 95% CI: − 3–25), or VO_2_ (1.3 ml·kg^−1^·min^−1^, 95% CI: − 0.2–3.5). ICC values were considered poor regardless of the measured variable (ICC ≤ 0.43). Bland–Altman analysis for the first threshold is shown in Fig. 1. A mean bias of 7 ± 16 bpm, 11 ± 31 W, and 1.8 ± 4.1 ml·kg^−1^·min^−1^ was found, while limits of agreement ranged from –25 to 39 bpm, − 50 to 72 W, and − 6 to 10 ml·kg^−1^·min^−1^ for HR, PO, and VO_2_ data, respectively.

Concerning the second threshold, all the correlation coefficients between VT2 and HRVT2 reached statistical significance (*p* ≤ 0.01). Strong correlations were found for HR (*r* = 0.88), PO (*r* = 0.82), and VO_2_ (*rho* = 0.88). Mean differences were not statistically significant for HR (− 2, 95% CI: − 6–3), PO (− 6 W, 95% CI: − 15–3), or VO_2_ (− 0.7 ml·kg^−1^·min^−1^, 95% CI: − 1.6–0.7). ICC values demonstrated good reliability for all variables: HR (ICC = 0.85), PO (ICC = 0.81), and VO_2_ (ICC = 0.86). Bland–Altman analysis for the second threshold is shown in Fig. 1. A mean bias of − 2 ± 10 bpm, − 11 ± 20 W, and − 0.5 ± 2.4 ml·kg^−1^·min^−1^ was found, while limits of agreement ranged from –21 to 18 bpm, − 44 to 3 W, and − 5 to 4 ml·kg^−1^·min^−1^ for HR, PO, and VO_2_ data, respectively.

## Discussion

The aim of this study was to assess the level of agreement between ventilatory and HRV thresholds in patients with CHF. Our initial hypothesis suggested a strong agreement between VT1 and HRVT1, based on a previous study conducted with cardiac patients, most of them with coronary artery disease (Rogers et al. 2021d) and that HRVT2 would show at least moderate concordance with VT2. The findings partly confirmed this hypothesis: HRVT2 displayed strong agreement with VT2 across all outcome variables, whereas HRVT1 achieved only low to moderate correlations with VT1.

### Agreement between HRVT1 and VT1

Regarding the first threshold, correlations between HRVT1 and VT1 varied across the measured variables. A non-significant, low correlation was observed for HR, while PO and VO_2_ showed significant low and moderate correlations, respectively. Despite these associations, the limits of agreement were wide for all variables. These findings contrast with those of Rogers et al. (2021d), who reported significant and strong correlations (*r* = 0.86 in HR, *r* = 0.87 in PO, *r* = 0.95 in VO_2_) with lower mean biases (3 ± 7 bpm, 5 ± 13 W, and 1.2 ± 2.9 ml·kg^−1^·min^−1^). Nevertheless, they presented wider limits of agreement (− 11 to 18 bpm, − 20 to 31 W, and − 4.6 to 7 ml·kg^−1^·min^−1^). Despite these differences, both studies consistently found that HRVT1 values were higher than VT1 values. While the limits of agreement between HRVT1 and VT1 remained wide, they are comparable to the ranges observed in studies comparing ventilatory and lactate thresholds (Pallarés et al. 2016) suggesting that such variability is consistent with standard threshold identification methods. Such disparities could stem from methodological differences, particularly in the determination of VT1. For instance, Rogers et al. (2021d) utilised the excess CO_2_ method with unaveraged breath-by-breath data, whereas we adopted a mixed method using averaged ventilatory data. Moreover, differences in patient populations could also contribute to these discrepancies. In the cited study, most participants had coronary artery disease, a condition that generally represents a less advanced stage of cardiovascular dysfunction compared to CHF, what may have influenced the results.

Additionally, differences in ECG recording devices might have influenced results. Rogers et al. (2021d) used a device with a lower sampling rate of 200 Hz, while our study employed an 8000 Hz sampling rate. Previous research has shown significant variations in DFA a1 values at rest when comparing devices with different sampling rates. Specifically, lower sampling frequencies introduce greater errors in RR interval detection, which can affect HRV measurements, particularly when HRV values are low (Tapanainen et al. 1999).

The use of beta-blockers by patients in this study may have further influenced the relationship between HRVT1 and VT1. Beta-blockers primarily block beta-adrenergic receptors, thereby limiting heart’s response to increases in circulating catecholamines without necessarily reducing sympathetic outflow (Hori et al. 1991). Because DFA a1 thresholds depend on the dynamic interaction between sympathetic and parasympathetic branches, this pharmacological modulation may have resulted in a blunted chronotropic response, potentially affecting the accuracy of HRVT1 estimations. Although parasympathetic withdrawal remains a key factor influencing HRV during exercise, the attenuated decline in HRV caused by partial beta-adrenergic blockade could still lead to a weaker correlation with VT1 (Ishida et al. 1997). In a recent randomized, placebo-controlled trial, acute cardioselective beta1-blockade (bisoprolol) in healthy adults had no significant impact on ventilatory efficiency or the ventilatory threshold, suggesting that the effect may be limited or population-specific (Forton et al. 2022). In this study, tests were scheduled in the afternoon to minimise acute effects from morning doses; nevertheless, residual activity due to variable half-lives and individual responses may persist. Finally, differences in the testing times across studies, such as in Rogers et al. (2021d), could contribute to variability in reported correlations.

### Agreement between HRVT2 and VT2

In contrast to HRVT1, HRVT2 showed stronger agreement with VT2. To the best of our knowledge, this is the first study to evaluate HRVT2 in cardiac population, specifically in patients with CHF. Our results indicate strong correlations between both methods, with the strongest agreement observed in VO_2_ and HR values. These results align with prior studies that evaluated this threshold in healthy populations. For example, Rogers et al. (2021c) reported similar findings in recreational male runners during an incremental treadmill test in HR values determined by the two methods (*r* = 0.78; mean bias − 4 ± 10 bpm, limits of agreement from − 24 to 16 bpm). Likewise, Schaffarczyk et al. (2022) reported even stronger correlation in a female cohort (Pearson’s *r* = 0.90; mean bias 0.5 ± 5.7 bpm). Additionally, Sempere-Ruiz et al. (2024) found similar results when examining HRVT2 in a population of healthy participants, reporting correlations exceeding *r* = 0.80 and narrower limits of agreement compared to HRVT1. These findings further reinforce the potential utility of the DFA a1 method as a reliable approach for determining the second threshold in patients with CHF. This is particularly relevant for exercise prescription in this population, as high-intensity aerobic training (above the second threshold) has been shown to be highly effective in CHF patients, improving cardiorespiratory fitness, vascular function, and overall prognosis (Turri-Silva et al. 2021; Fuertes-Kenneally et al. 2023).

Mechanistically, the correlation between DFA a1 and ventilatory thresholds may be explained by their shared reflection of systemic physiological responses to exercise. This stage marks a progressive transition in the relative contribution of metabolic pathways, with a gradual increase in anaerobic glycolysis as exercise intensity rises, which are accompanied by changes in ANS activity. DFA a1, as a non-linear HRV metric, is sensitive to alterations in ANS regulation, particularly the balance between sympathetic and parasympathetic inputs. Rogers et al. (2021c) proposed that the progressive loss of fractal-like properties in HRV, reflected in decreases in DFA a1, corresponds to increased physiological stress and the body's struggle to maintain homeostasis at higher exercise intensities. This conceptual overlap suggests that DFA a1 can serve as a surrogate marker for ventilatory thresholds, providing a cost-effective and accessible method for exercise intensity prescription.

### Clinical applications

HRVT2 showed good agreement with VT2, supporting its potential as a practical surrogate for defining the upper boundary of the heavy-intensity domain in cardiac-rehabilitation settings where CPET is unavailable, enhancing the precision of exercise prescription. This perspective aligns with the concerns highlighted by Keir (2024) regarding the challenges and limitations of CPET in clinical practice, such as cost, accessibility, and patient burden. In contrast, HRVT1 displayed only modest correspondence with VT1. Until larger CHF cohorts confirm its validity, HRVT1 should be used with caution and preferably in combination with other sub-maximal markers.

Nonetheless, the operational simplicity of DFA a1 calculation—single-channel RR recording from a HR monitor or ECG, and fixed cutoff values for HRVT1 (0.75) and HRVT2 (0.5) that are presumed to remain stable regardless day-to-day training and fatigue—remains attractive. Clinicians could employ HRVT2 to individualise interval workloads, to verify whether a bout remained below or above the second threshold, or to adjust home-based programmes without previous laboratory gas analysis, providing a promising framework for real-time monitoring and individualised adjustment of training intensity.

### Study limitations and future research

Several limitations must be acknowledged. First, because no a priori sample-size estimation was performed, we cannot ascertain whether the study was adequately powered to detect smaller between-method differences. However, differences between the two methods were estimated as a measure of effect size magnitude. Second, a high number of patients were excluded from the analysis due to difficulties in detecting first and second thresholds. This challenge is common in studies employing CPET in clinical populations. Keir (2024) highlighted that the identification of ventilatory thresholds in patients with cardiac pathologies is often compromised by individual physiological variability, methodological inconsistencies, and technical limitations. Furthermore, these factors may be exacerbated in CHF patients, where disease-related alterations in ventilatory and cardiovascular responses further complicate the accurate determination of thresholds. Consequently, the observed data loss in our study aligns with previously reported challenges in applying CPET-derived thresholds in clinical settings, reinforcing the need for alternative, more accessible methods for exercise intensity prescription. Third, our sample was predominantly male (65%), and the small number of women (*n* = 7) precluded a statistically powered sex-stratified analysis. Future studies with larger and more balanced cohorts are required to determine whether the present findings generalise equally to males and females. Finally, all participants were stable NYHA class II–III heart-failure patients treated according to contemporary guidelines; caution is warranted when extrapolating the results to other heart-failure phenotypes or to patients with more advanced functional limitation.

Future research should aim to validate these findings in larger, more diverse cohorts and explore the longitudinal applicability of HRV thresholds in CHF management. Additionally, it should focus on validating the use of DFA a1 across a broader range of populations and pathologies, including individuals with other chronic conditions such as chronic obstructive pulmonary disease or metabolic syndrome. Additionally, further investigation is needed to confirm the reliability of DFA a1 in delineating exercise intensity domains during prolonged and repeated sessions. This includes exploring how factors like fatigue and long-term training adaptations might influence exercise intensity thresholds. Combining DFA a1 with complementary methods, such as near-infrared spectroscopy, could also enhance the precision of exercise monitoring by providing a more comprehensive view of systemic and local muscle oxygenation responses. Ultimately, these efforts will help establish DFA a1 as a versatile and widely applicable tool for exercise prescription and monitoring.

## Conclusion

Our study revealed that, in patients with CHF, HRVT2 exhibited very good agreement with VT2 across all measured variables. Thus, a DFA a1 value of 0.50 may serve as a practical, low-cost surrogate for VT2 when CPET is unavailable, and, therefore, a useful anchor for exercise intensity prescription in resource-limited settings.

By contrast, HRVT1 correlated only modestly with VT1 and displayed wide limits of agreement, suggesting its applicability remains limited in this population. Until larger studies clarify whether protocols adjustments improve precision, HRVT1 should be considered cautiously and in conjunction with traditional sub-maximal anchors.

Overall, the present data support incorporating HRVT2—as derived from simple RR-interval recordings—into cardiac-rehabilitation practice as a complementary tool that enhances individualisation and accessibility of exercise prescription, while additional work is needed before HRVT1 can be recommended for routine use in heart-failure rehabilitation.

## Data Availability

The data underlying this article will be shared on reasonable request to the corresponding author.

## Abbreviations

95%CI: 95% Confidence interval
ACE: Angiotensin-converting enzyme
ANS: Autonomic nervous system
BMI: Body mass index
CHF: Chronic heart failure
CPET: Cardiopulmonary exercise test
CR: Cardiac rehabilitation
DFA a1: Alpha 1 of detrended fluctuation analysis
ECG: Electrocardiography
HR: Heart rate
HRV: Heart rate variability
HRVT: Heart rate variability threshold
HRVT1: First heart rate variability threshold
HRVT2: Second heart rate variability threshold
ICC: Intraclass correlation coefficient
ICD: Implantable cardioverter defibrillator
LVEF: Left ventricular ejection fraction
MRA: Mineralocorticoid receptor antagonists
PO: Power output
SD: Standard deviation
VO₂: Oxygen uptake
VT: Ventilatory threshold
VT1: First ventilatory threshold
VT2: Second ventilatory threshold
